# Case Report: Challenging Treatment of an AorticParavalvular Leak: How We Avoided Interference With Mechanical Valve Function?

**DOI:** 10.3389/fcvm.2022.839159

**Published:** 2022-06-27

**Authors:** Eustaquio Maria Onorato, Matteo Vercellino, Annamaria Costante, Antonio L. Bartorelli

**Affiliations:** ^1^Centro Cardiologico Monzino, IRCCS, Milan, Italy; ^2^Cardiology Department, IRCCS, Ospedale Policlinico San Martino, Genova, Italy; ^3^Cardiology Department, Azienda Ospedaliera di Alessandria, Alessandria, Italy; ^4^Department of Biomedical and Clinical Sciences Luigi Sacco, University of Milan, Milan, Italy

**Keywords:** paravalvular leak, aortic valve, transcatheter closure, hemolytic anemia, heart failure

## Abstract

**Background:**

Aortic paravalvular leak (APVL) after surgical valve replacement (AVR) is an ominous complication with a high risk of morbidity and mortality. Approximately 1–5% of PVLs can lead to serious clinical consequences, including congestive heart failure and/or hemolytic anemia.

**Case Summary:**

A 69-year-old man with multiple comorbidities underwent surgical replacement of the aortic valve with a mechanical tilting disc prosthetic valve (Medtronic Starlight 27 mm). Several years later, recurrent episodes of congestive heart failure and hemolytic anemia developed due to a large crescent-shaped aortic PVL located at non coronary cusp (NCC) 9–12 o'clock, with moderate-to-severe regurgitation. The patient was deemed at prohibitive surgical risk due to significant multiple comorbidities and a transcatheter PVL closure (TPVLc) was planned. The huge PVL was partially closed by a first specifically designed paravalvular leak device (PLD). The procedure was complicated by transient interference of the second PLD with mechanical prosthetic valve function. This issue has however been solved with correct manipulation, orientation and downsizing of the second device implanted. At 3-month and 13-month follow-up, the patient showed a relevant clinical improvement and good quality of life. 2D TTE color Doppler confirmed the stable position of the two PLDs with trace residual leak.

**Discussion:**

Surgical redo has been considered the treatment of choice for symptomatic patients with PVLs. Notwithstanding, TPVLc is a less invasive alternative, particularly in patients at high surgical risk in whom early diagnosis and prompt interventional treatment are crucial for improving expectancy and quality of life. Dedicated devices, appropriate procedural techniques, and the close interaction between imaging modalities, allowed to deal successfully with a challenging case of severe symptomatic aortic PVL.

## Introduction

Paravalvular leaks occur in patients who have undergone surgical valve replacement, with an incidence of 2–10% in the aortic position. Although most defects are small and asymptomatic, clinical manifestation of heart failure and hemolysis can be demonstrated in 1%-3% of patients, which leads to worst outcome and justify an interventional approach (1).

For patients with symptomatic PVL, surgical repair is the traditional treatment approach. Surgical results have shown to significantly improve patient outcomes at 5 and 10 years compared to medical treatment alone (2), despite the significant mortality associated with re-operation (3).

TPVLc is a less invasive treatment option, particularly in patients with prohibitive risk for redo surgery in whom it may represent a first-line treatment.

The safety and feasibility of TPVLc has been confirmed in several registries and a meta-analysis (4, 5). Overall technical success rate was 89.7%, being comparable between patients treated for mitral or aortic valve PVLs (92.6 vs. 83.3%, respectively) (6).

Nevertheless, catheter-based interventions are associated with higher rates of residual leaks in part due to complex anatomies, size and number of defects and to off-label use of devices designed for other applications.

## Case Presentation

A 69-year-old male patient was admitted for recurrent episodes of congestive heart failure and hemolytic anemia. He has a past medical history of rheumatoid arthritis, Sjogren syndrome, moderate kidney disease (stage 3), chronic obstructive pulmonary disease, fronto-parietal left ischemic stroke with right side hemiparesis and rheumatic heart disease status following aortic valve replacement in 2005 with a mechanical tilting disc aortic prosthetic valve (Medtronic Starlight 27 mm). In the recent years, recurrent hospital admissions for heart failure and symptomatic hemolytic anemia requiring multiple red blood cells transfusions concomitantly occurred. The patient was in New York Heart Association (NYHA) class III under optimal medical therapy (OMT) (furosemide 50 mg/day, spironolactone 25 mg/day, bisoprolol 2.5 mg/day, ACE inhibitors 20 mg/day, aspirin 100 mg/day, statin 20 mg/day). Frequent episodes of dyspnea at rest with orthopnea (NYHA class IV) occurred during hemolysis, documented by low haptoglobin level and lactate dehydrogenase >500 UI/L. After red blood cell transfusions, the clinical and hemodynamic conditions improved significantly each time. Electrocardiogram showed biatrial enlargement, left bundle branch block and frequent ventricular ectopic beats. During each episode of congestive heart failure, chest X-ray revealed signs of increased pulmonary flow, dilatation of left-sided chambers as well as enlargement of pulmonary artery and its branches. Baseline two-dimensional (2D) Transthoracic/Transesophageal color Doppler Echocardiography (TTE/TEE) showed severe left ventricle (LV) dilatation (LV end-diastolic volume of 216 ml) with ejection fraction of 50% and confirmed the presence of a large 17 × 6 mm crescent-shaped aortic PVL located at non coronary cusp (NCC) 9–12 o'clock, with grade 4–5 aortic PVL regurgitation (PHT < 200 msec; vena contracta width = 10 mm) (7). Coronary angiography excluded coronary artery disease.

**Figure 1:**
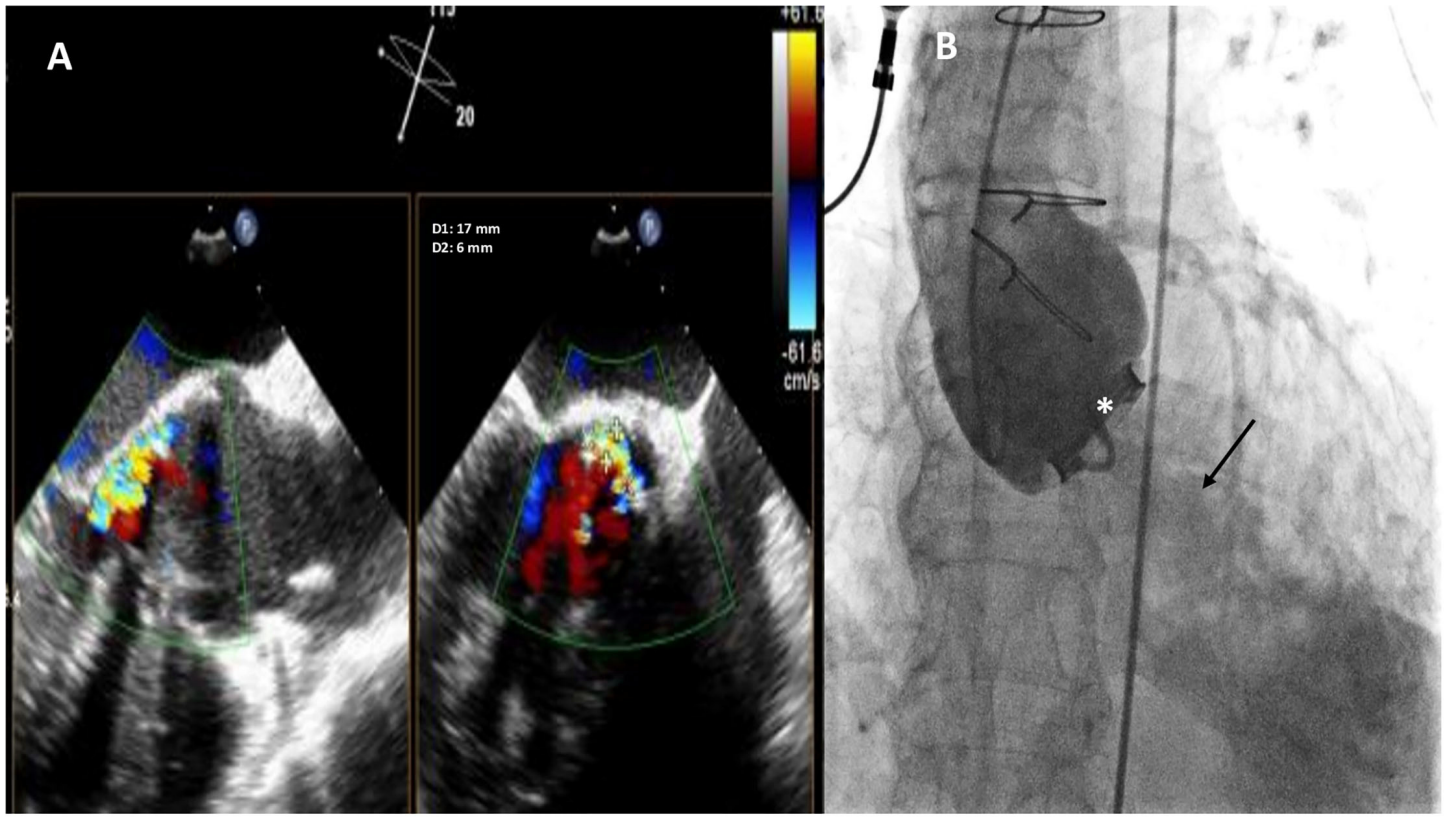
Baseline two-dimensional (2D) X-plane imaging Transoeophageal Echocardiogram (TEE) color Doppler **(A)** and ascending aorta angiography **(B)** showing the presence of a huge crescent-shaped 17 × 6 mm aortic PVL located at non coronary cusp (NCC) 9-12 o'clock, with moderate-to-severe regurgitation. Fluoro-angiographic image confirming the stable position of the mechanical tilting disc aortic prosthetic valve (white asterisk) with moderate regurgitation (black arrow).

**Figure 2:**
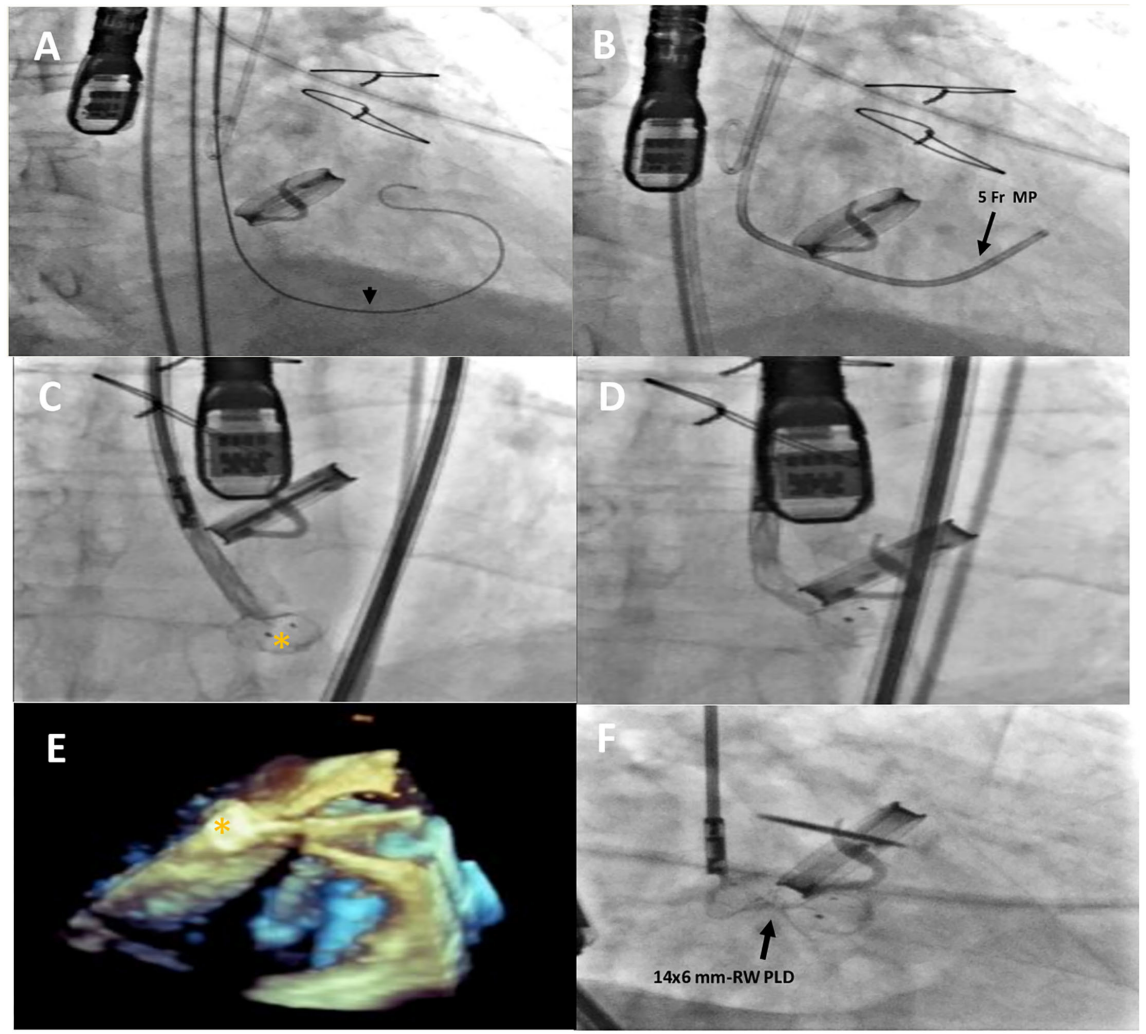
Intraprocedural fluoro-angiographic and real-time 3D TEE procedural steps showing the guide wire **(A)** (black arrowhead) across the leakage with the distal soft tip in the left ventricle; the 5- Fr multipurpose catheter in the LV **(B)**; the distal disc device opening (orange asterisk) **(C–E)** and the 14 × 6 mm rectangular waist PLD correctly positioned and still anchored to the delivery cable **(F)**. MP, multipurpose catheter; PLD, Occlutech Parvalvular Leak Device; RW, rectangular waist.

**Figure 3:**
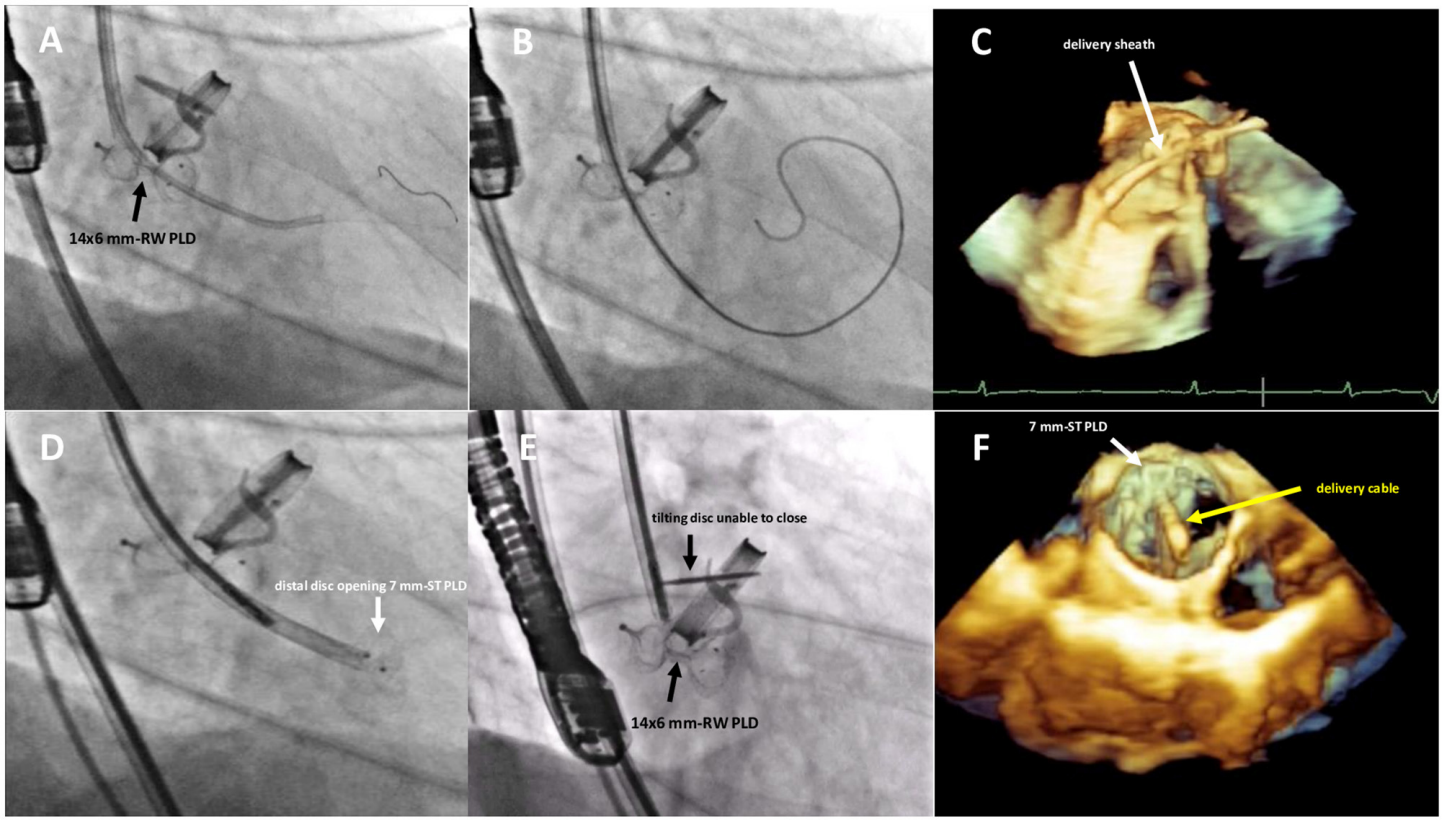
Fluoro-angiographic and real-time 3D TEE procedural steps showing the crossing of the residual leakage in close proximity of the already implanted 14 × 6 mm rectangular waist PLD **(A–C)**, the distal disc opening of the 7 mm square twist PLD **(D)** still anchored to the delivery cable and the inference with the tilting disc of the mechanical aortic valve **(E,F)**. RW, rectangular waist; ST, square twist.

**Figure 4:**
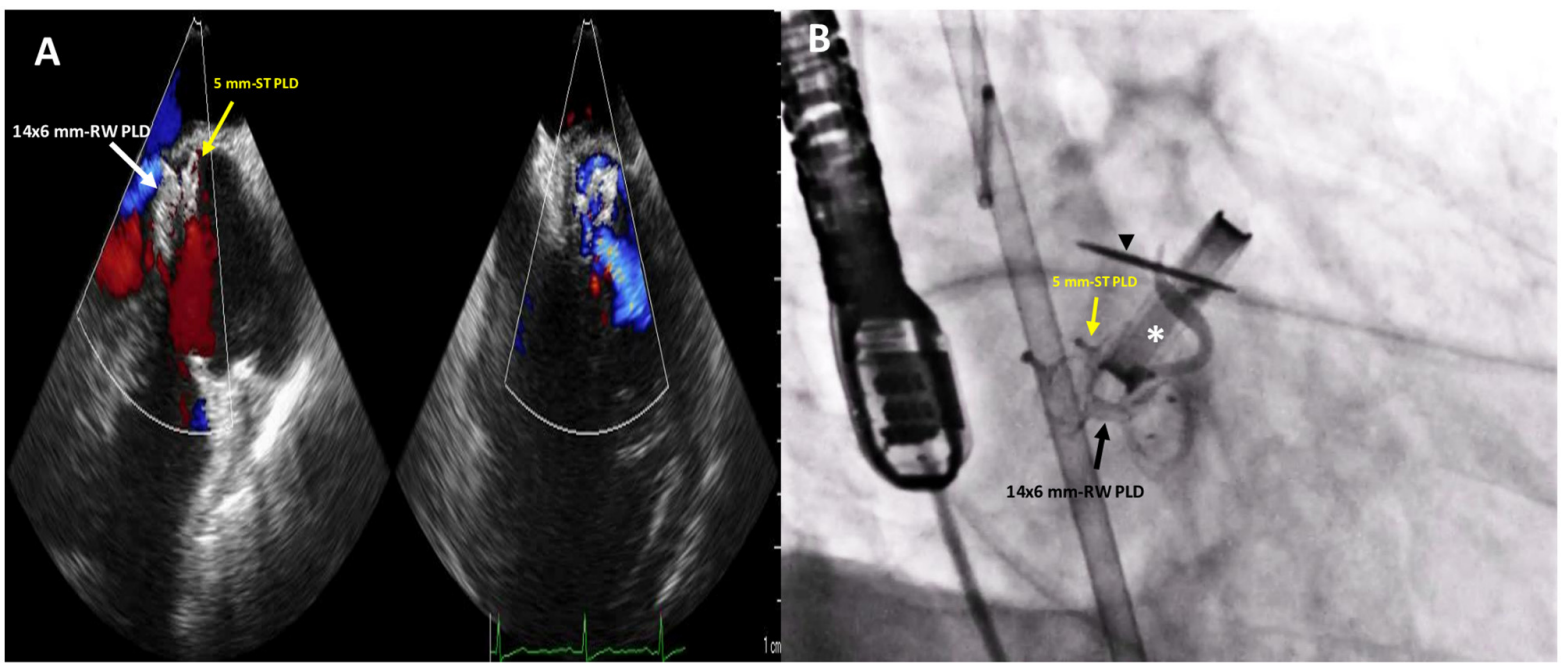
Post-procedure 2D TTE color Doppler **(A)** and fluoro-angiographic **(B)** images showing the correct and stable position of the two PLDs not impinging on the tilting disc (black arrowhead) of the mechanical aortic valve (white asterisk) with only trivial residual regurgitant jet.

The risk stratification according to Society of Thoracic Surgeons (STS) score demonstrated an increased risk of long-term mortality (7%). According to 2014 AHA/ACC guidelines, TPVLc was a reasonable therapeutic option (class IIa, Level of Evidence B) in centers with expertise in symptomatic patients at high risk for surgical reintervention or with contraindication to reoperation (8). After heart team discussion, the decision to proceed with TPVLc was confirmed. Written informed consent, after explanation, was obtained from the patient. The procedure was performed under TEE and fluoroscopic guidance using general anesthesia. Prevention of contrast induced nephropathy was accomplished with normal saline 0.9% infusion (40 ml/h 12 h before and after procedure). Both left and right femoral arteries were cannulated. 4–5 degree paravalvular aortic regurgitation was demonstrated by 2D TEE color Doppler and by ascending aortography with a 6-Fr pigtail angiographic catheter introduced from the left femoral artery (Figure 1). With the support of a 5-Fr multipurpose catheter (MP), introduced retrogradely from the right femoral artery, we easily advanced and passed through the huge NCC leakage a hydrophilic Terumo 0.035 inch guide wire, then replaced by a super stiff guidewire 0.035-inch 260 cm in lenght (Boston Scientific) that was positioned in the distal LV. The stronger support allowed progression of the 10-Fr delivery sheath into the LV. To accomplish the semilunar PVL dimensions (17 × 6 mm) with a height of 7 mm, a specifically designed 14 × 6 mm rectangular waist Paravalvular Leak Device (PLD, Occlutech, Helsingborg, Sweden) with a 24 mm distal disc, a 22 mm proximal disc and a 3 mm-height waist type of connection between the two disc was chosen and deployed (Figure 2). Intraprocedural 2D TEE color Doppler showed a persisting regurgitant jet via a 10 × 4 mm residual peri-device leakage probably due to the distorted “mushroom” spherical configuration of the two device disc and to elongated (up to 7 mm) shape of the connecting waist to fit the height of the original PVL (Supplementary Figure 1), hindering the apposition of the fabric within the disc. At this point, with the first PLD still anchored, the residual peri-device leak was crossed again with a hydrophilic guide wire, allowing the progression of a 5-Fr MP. A 0.035-inch wire 260 cm super stiff guidewire was advanced distally via the MP into the LV. The same 10-Fr delivery sheath was advanced over this exchange stiff wire allowing the progression of a second device, a 7 mm square twist PLD. After its opening, a massive intra-prosthetic aortic regurgitation was observed by the TEE monitoring. The angiogram revealed an acute interference between the 7 mm square twist PLD and the prosthetic valve, showing the tilting disc unable to close completely. Therefore, the 7 mm square twist PLD was then retrieved in the sheath and replaced by an undersized 5 mm square twist PLD that was implanted effectively without interfering anymore with the tilting disc of the mechanical aortic valve (Figure 3). Post-procedure echocardiographic and angiographic controls confirmed the effective PVL closure with only trivial residual regurgitant jet (Figure 4). The total volume of contrast medium was 80 cc. Peak creatinine level was 2.12 mg/dl 48 h after the procedure and 1.6 mg/dl at dismission, leading to the diagnosis of stage 1 acute kidney injury. The postoperative course was uneventful, and the patient was discharged on the eighth post-procedure day. At three-month and thirteen-month follow-up, he showed a relevant clinical improvement with a stable NYHA II class and good quality of life. No further hospitalizations and blood transfusions were needed. 2D TTE color Doppler confirmed the stable position of the two PLDs with trace residual leak (Supplementary Figure 2).

## Discussion

Comparison between surgical and percutaneous treatment have shown that technical success was higher with surgery [96.7 vs. 72.1%, odds ratio (OR) 9.7, *p* < 0.001] but at the cost of higher 30-day mortality (8.6 vs 6.8%, OR 1.90, *p* < 0.001), a trend toward higher stroke (3.3 vs. 1.4%, OR 1.94, *p* = 0.069), and longer hospitalizations. However, surgery was associated with similar 1-year mortality (17.3 vs. 17.2%, OR 1.07, *p* = 0.67), reoperation (9.1 vs. 9.9%, OR 0.72, *p* = 0.1), readmission for heart failure (13.3 vs. 26.4%, OR 0.51, *p* = 0.29), and improvement in New York Heart Association classification (67.4 vs. 56%, OR 1.37, *p* = 0.74), compared with percutaneous closure (9). Furthermore, PVL recurrence after first redo surgery has been reported to be 13% and increases further to 35% after second redo surgery (10). Reintervention rates were similar (11.3 vs. 17.2% in the percutaneous and surgical groups, respectively; *p* = 0.10), with the majority of reinterventions in the percutaneous group occurring early because of residual leak or persistent hemolysis. After risk adjustment, there was no significant difference in long-term survival between patients who underwent surgical vs. transcatheter treatment of PVLs (10).

TPVLc is a reasonable alternative to surgical intervention but it might be considered in patients with severe prosthetic paravalvular regurgitation with intractable hemolysis or NYHA class III or IV symptoms, who are at high or prohibitive surgical risk and have anatomical features suitable for percutaneous repair (11).

The efficacy of TPVL closure in reducing heart failure symptoms and long-term mortality has been demonstrated in multiple studies (12). Conversely, an incomplete TPVL closure could represent a very important limitation of this procedure, causing a worsening of hemolysis and NYHA class.

Undoubtedly, TPVL closure remains a complex, technically demanding and time-consuming procedure that requires not only careful patient selection and preprocedural multiple imaging modalities in order to visualize the complex 3D relationships of intracardiac structures. Additionally, it requires an active and strong collaboration of a skilled interventional team (interventional and imaging cardiologists, cardiac computed tomography radiologists and cardiac surgeons when needed).

Efforts are needed to develop new specifically designed devices to further improve clinical outcomes of this particular subset of high-risk patients with multiple comorbidities (13).

Even using a specific designed device, potential complications such as device malpositioning and interference between PLD and mechanical prosthetic valve may occur as demonstrated by our case. However, awareness of the pros and cons of each device, correct handling integrated with the echocardiographic imaging and good understanding of some procedural tips and tricks, could improve significantly the procedural success and clinical outcomes.

## Conclusion

Our case demonstrates that even though TPVLc with a specifically designed device is a viable therapeutic alternative to surgical repair with high technical success rate, a close collaboration of a skilled interventional team is key for success, avoiding potential procedural complications such as interference with mechanical valve function and incomplete leakage closure that may increase NYHA class, the magnitude of hemolysis and the need for hemolysis-related blood transfusions.

## Author's Note

This paper was the original work of the authors who have all seen and approved of the paper and authorship. The article has not been published elsewhere and is not under consideration in any other journals.

## Data Availability Statement

The original contributions presented in the study are included in the article/Supplementary Material, further inquiries can be directed to the corresponding author.

## Ethics Statement

The studies involving human participants were reviewed and approved by Centro Cardiologico Monzino, IRCC, Milan, Italy. The patients/participants provided their written informed consent to participate in this study.

## Author Contributions

EO: conceptualization, writing—original draft preparation, visualization, and validation. MV and EO: methodology and data curation. AC: software. AB and EO: formal analysis, writing—review and editing, supervision, and investigation. All authors have read and agreed to the published version of the manuscript.

## Conflict of Interest

EO is a consultant for Occlutech, manufacturer of the device. The remaining authors declare that the research was conducted in the absence of any commercial or financial relationships that could be construed as a potential conflict of interest.

## Publisher's Note

All claims expressed in this article are solely those of the authors and do not necessarily represent those of their affiliated organizations, or those of the publisher, the editors and the reviewers. Any product that may be evaluated in this article, or claim that may be made by its manufacturer, is not guaranteed or endorsed by the publisher.

## Supplementary Material

Supplementary Figure 1 Post-procedure 2D X-plane **(A)** and short axis view **(B)** TEE color Doppler showing a regurgitant jet through a 10 × 4 mm residual peri-device leakage. RW, rectangular waist; PLD, Occlutech Parvalvular Leak Device.

Supplementary Figure 2 Three-month follow-up 2D TTE **(A)** color Doppler **(B)** confirmed the stable position of the two PLDs with trace residual leak. PLD, Occlutech Paravalvular Leak Device.
