# Supplementary figures and images for: Case Report: Challenging Treatment of an AorticParavalvular Leak: How We Avoided Interference With Mechanical Valve Function?
Source: Front Cardiovasc Med. 2022 Jun 27;9:839159. doi: 10.3389/fcvm.2022.839159 (PMC9272523; doi:10.3389/fcvm.2022.839159)

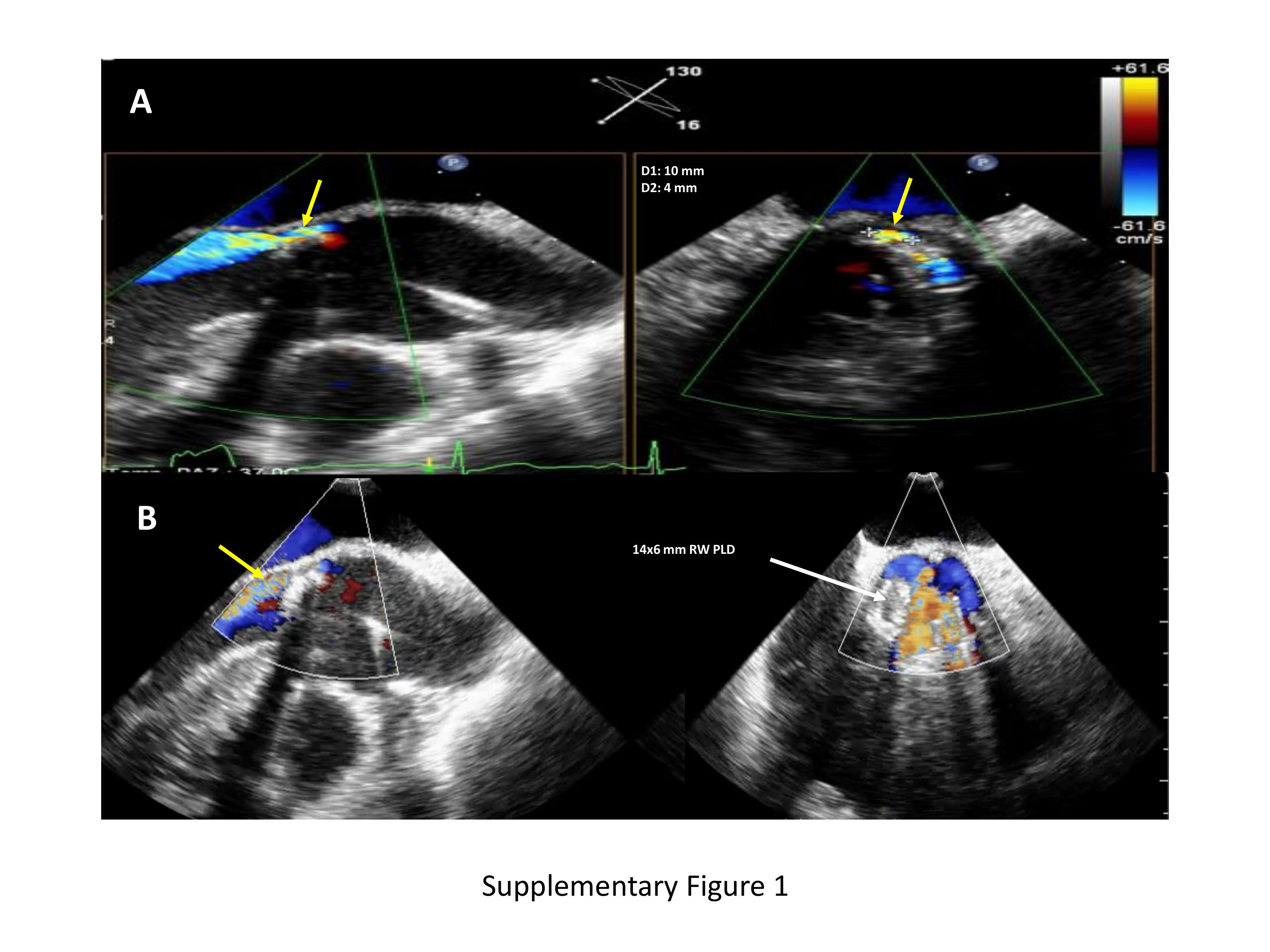

Supplement: Supplementary Figure 1 — Post-procedure 2D X-plane (A) and short axis view (B) TEE color Doppler showing a regurgitant jet through a 10 × 4 mm residual peri-device leakage. RW, rectangular waist; PLD, Occlutech Parvalvular Leak Device. [file Image_1.jpeg]

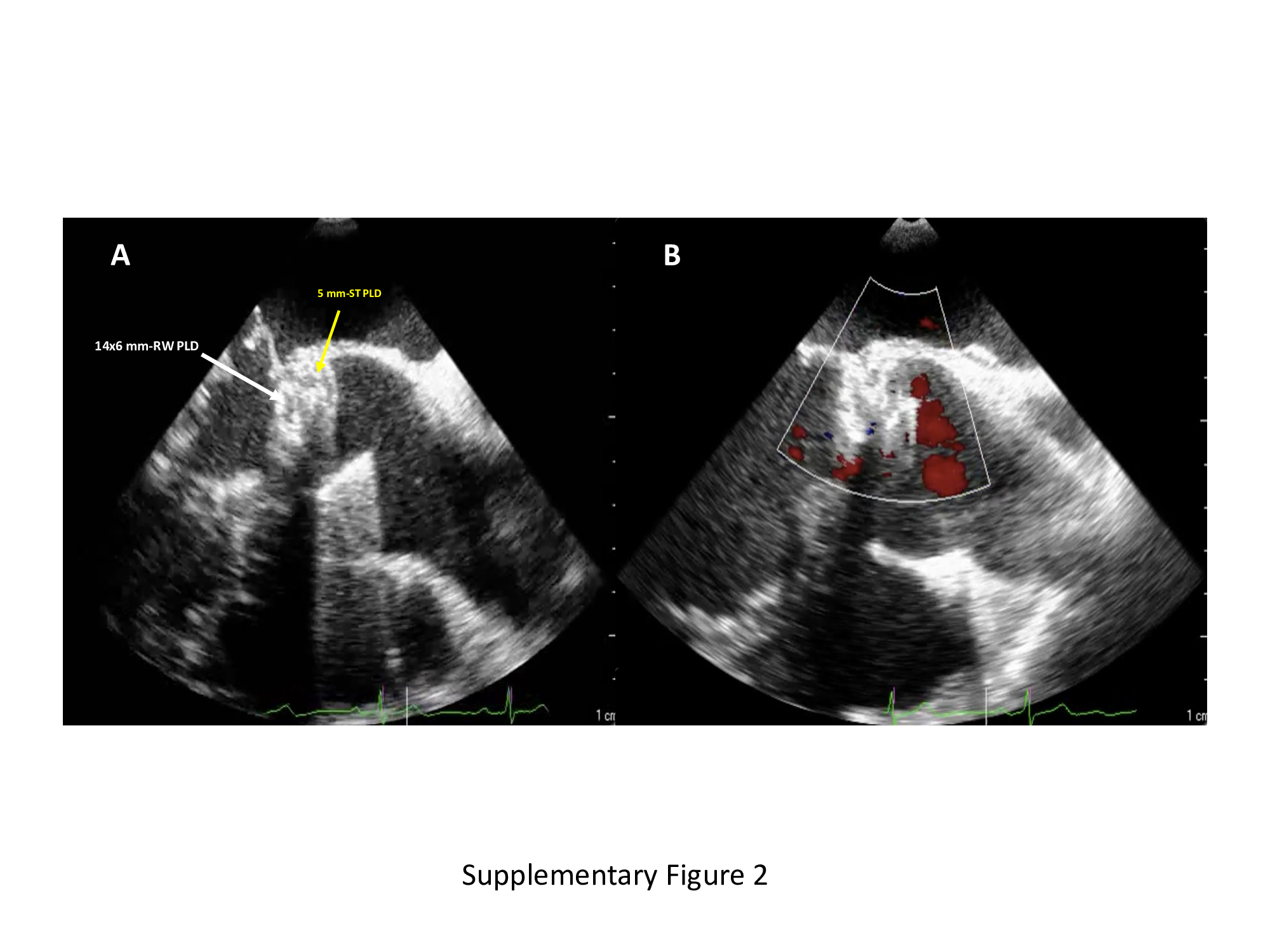

Supplement: Supplementary Figure 2 — Three-month follow-up 2D TTE (A) color Doppler (B) confirmed the stable position of the two PLDs with trace residual leak. PLD, Occlutech Paravalvular Leak Device. [file Image_2.jpg]
